# Bispecific antibodies enhance tumor‐infiltrating T cell cytotoxicity against autologous HER‐2‐expressing high‐grade ovarian tumors

**DOI:** 10.1002/JLB.5MA1119-265R

**Published:** 2019-12-13

**Authors:** Hans‐Heinrich Oberg, Lisa Janitschke, Vjola Sulaj, Jörg Weimer, Daniel Gonnermann, Nina Hedemann, Norbert Arnold, Dieter Kabelitz, Matthias Peipp, Dirk Bauerschlag, Daniela Wesch

**Affiliations:** ^1^ Institute of Immunology Christian‐Albrechts University of Kiel Kiel Germany; ^2^ Department of Gynecology and Obstetrics UKSH Campus Kiel Kiel Germany; ^3^ Institute of Clinical Molecular Biology UKSH CAU Kiel Kiel Germany; ^4^ Division of Stem Cell Transplantation and Immunotherapy, Department of Medicine II UKSH CAU Kiel Kiel Germany

**Keywords:** bispecific antibody, cisplatin, HER‐2, human γδ T cells, ovarian cancer, T cell subsets

## Abstract

Epithelial ovarian cancer displays the highest mortality of all gynecological tumors. A relapse of the disease even after successful surgical treatment is a significant problem. Resistance against the current platinum‐based chemotherapeutic standard regime requires a detailed ex vivo immune profiling of tumor‐infiltrating cells and the development of new therapeutic strategies.

In this study, we phenotypically and functionally characterize tumor cells and autologous tumor‐derived αβ and γδ T lymphocyte subsets. Tumor‐infiltrating (TIL) and tumor‐ascites lymphocytes (TAL) were ex vivo isolated out of tumor tissue and ascites, respectively, from high‐grade ovarian carcinoma patients (FIGO‐stage IIIa‐IV). We observed an increased γδ T cell percentage in ascites compared to tumor‐tissue and blood of these patients, whereas CD8^+^ αβ T cells were increased within TAL and TIL. The number of Vδ1 and non‐Vδ1/Vδ2‐expressing γδ T cells was increased in the ascites and in the tumor tissue compared to the blood of the same donors. Commonly in PBL, the Vγ9 chain of the γδ T cell receptor is usually associated exclusively with the Vδ2 chain. Interestingly, we detected Vδ1 and non‐Vδ1/Vδ2 T cells co‐expressing Vγ9, which is so far not described for TAL and TIL.

Importantly, our data demonstrated an expression of human epidermal growth factor receptor (HER)‐2 on high‐grade ovarian tumors, which can serve as an efficient tumor antigen to target CD3 TIL or selectively Vγ9‐expressing γδ T cells by bispecific antibodies (bsAbs) to ovarian cancer cells. Our bsAbs efficiently enhance cytotoxicity of TIL and TAL against autologous HER‐2‐expressing ovarian cells.

## Graphical abstract

Tumor‐infiltrating γδ T cells are attractive effector cells for targeting with bispecific antibodies in the treatment of ovarian cancer cells.

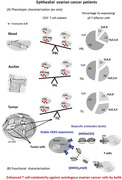

**Abbreviations**
bsAb: bispecific antibody
EpCAM: epithelial cell adhesion molecule
HER‐2: human epidermal growth factor receptor
KI‐OC: Kiel‐Ovarian Cancer cells
RTCA: real time cell analyzer
TAL: tumor‐ascites lymphocytes
TCR: T cell receptor
TIL: tumor‐infiltrating lymphocytes

## 1 INTRODUCTION

Epithelial ovarian cancer causes around 140,000 deaths annually in women worldwide. With a 5‐yr‐survival rate of only <40% after first‐line therapy ovarian cancer remains a highly lethal tumor entity to this day.^1^ Due to the absence of initial symptoms the disease is often diagnosed at an advanced stage accompanied with an accumulation of ascites fluid in the peritoneal cavity, which facilitates the dissemination of tumor cells in the surrounding microenvironment.^2^ The standard therapy is a surgical resection followed by platinum‐based therapy. A significant problem for an effective treatment is the recurrence of the disease even after successful surgical treatment—caused in many cases by a resistance against the current platinum‐based chemotherapeutic standard regimen.^3^ Many attempts to improve ovarian cancer patients’ outcome by adding additional drugs failed. Trastuzumab treatment in ovarian cancer is not established partially due to the high tumor heterogeneity, which leads to lower response rates.^4^ Combining chemotherapy with the humanized human epidermal growth factor receptor (HER)‐2 mAb trastuzumab has been described in a few clinical trials to improve overall patients’ survival compared to chemotherapy alone in HER‐2‐expressing uterine serous and high‐grad endometrioid tumors.^4^, ^5^, ^6^ Commonly, HER‐2 expression is detected in 22–66% of ovarian cancer patients.^7^ New therapeutic strategies such as the development of bispecific antibodies (bsAbs) with the aim to achieve more effective and durable response, are of major clinical interest.^8^, ^9^, ^10^ The bsAbs enhance cytotoxic activity by selectively targeting immune cells to tumor‐associated antigens such as HER‐2.^11^, ^12^, ^13^ Recently, we demonstrated an enhanced lysis of ex vivo isolated ovarian tumor cells as well as of cisplatin‐resistant SK‐OV‐3 cells by activating γδ T cells of peripheral blood lymphocytes (PBL) or tumor tissue via bsAbs.^14^ Human γδ T lymphocytes are attractive effector cells for T cell‐based immunotherapy due to their strong cytotoxic activity, antigen recognition in HLA‐independent manner, their reduced induction of a severe graft‐versus‐host disease, the capacity of Vδ2 γδ T cell subset to present antigens to αβ T cells, as well as their production of Th1 cytokines, which could compensate for the reduced number of Th1‐producing CD4 T cells at the tumor site.^15^, ^16^

γδ T cells can be divided into two main subsets, the T cell receptor (TCR) Vδ2 T cells, which predominate the blood and the TCR Vδ1 T cells representing the main subset in the tissue. Both subsets infiltrate in solid tumors and expand to an 800‐ to 1000‐fold increase after their activation.^13^, ^15^, ^17^ Vδ1 T cells recognize microbial and self‐lipids bound to nonclassical CD1d molecules as well as stress‐induced MHC class I‐related chain A/B (MICA/B) molecules, which are constitutively expressed on tumor cells.^18^, ^19^ Vδ2 T cells are specifically activated by pyrophosphate intermediates of the prokaryotic nonmevalonate pathway or a dysregulated mevalonate pathway of transformed eukaryotic cells.^20^, ^21^

In our study, we phenotypically characterized the TCRγ‐repertoire of γδ T cell subsets by immunophenotyping with in‐house anti‐TCRVγ mAb in ex vivo isolated tumor tissue compared to autologous blood and ascites of ovarian cancer. Importantly, the cytotoxic capacity of different αβ‐ and γδ T cell subsets isolated out of PBL, tumor‐ascites lymphocytes (TAL), and tumor‐infiltrating lymphocytes (TIL) from the same donor against autologous tumor cells were analyzed. Recent developed bsAbs were analyzed whether they target tumor‐derived T cell subsets to autologous ovarian tumor cells and enhance the cytotoxicity of these T cell subsets against ovarian cancer cells.

## 2 MATERIAL AND METHODS

### 2.1 Patient cohort

Heparinized blood, ascites, and tumor tissue from patients were obtained from the Department of Gynecology and Obstetrics of the University Hospital Schleswig‐Holstein (UKSH) in Kiel, Germany. Leukocyte concentrates from healthy adult blood donors were obtained from the Department of Transfusion Medicine of the UKSH in Kiel. Informed consent was obtained from all donors in accordance with the Declaration of Helsinki, and the research was approved by the relevant institutional review boards (ethic committee of the Medical faculty of the CAU to Kiel, code number: D 445/18).

For flow cytometry analysis, 31 patients with histologically verified advanced ovarian cancer (FIGO‐stage IIIA‐IV, age 59.1 ± 10.4 yr) were enrolled. 29 of the patients had not been treated with chemotherapy or radiotherapy prior to this investigation.

For functional assays, patients with serous ovarian cancer (FIGO‐stage IIIC, high grade, age 53.8 ± 9.9 yr) at first diagnosis, shortly after surgery and before chemotherapy were enrolled. Two patients (KI‐OC‐11, FIGO‐stage IIIC, high grade and KI‐OC‐37, FIGO‐stage IV, high grade) were treated with adjuvant therapies followed by 3 further cycles with carboplatin/paclitaxel before the time point of investigation. From KI‐OC‐11 tumor material and blood was obtained after a relapse of the disease.

### 2.2 Ex vivo isolation of peripheral blood‐, tumor‐derived lymphocytes, and tumor cells

Tumors of advanced ovarian cancer patients removed during surgery were dissected in tumor tissues by the pathologists of the UKSH. Tumor tissues (1–100 cm^3^) and ascites (50–100 mL, if available) were provided by the Department of Gynecology and Obstetrics, UKSH, Campus Kiel. Tumor tissues were washed (in 10 cm dishes) with PBS to remove blood debris. Subsequently, the tumor tissues were minced into approximately 1 mm^3^ pieces and treated with components A, H, and R of the Tumor Dissociation Kit (Miltenyi Biotec, Bergisch Gladbach, Germany) for 1 h at 37°C in 5 mL PBS in a gentle MACS (Miltenyi Biotec). Digested cell suspension was then passed through a 100 µm cell strainer (Falcon, BD Biosciences, Heidelberg, Germany), visually controlled by light microscopy and centrifuged at 481 ×*g* for 5 min. Tumor cells as well as TIL were isolated by Ficoll‐Hypaque (Biochrom, Berlin, Germany) density gradient centrifugation.

Ascites was centrifuged at 481 ×*g* for 5 min and supernatant was immediately collected after centrifugation and frozen at −20°C. Cell pellet was resuspended in complete medium and tumor cells and TIL were isolated by Ficoll‐Hypaque density gradient centrifugation.

TIL were immediately used for the different assays or for establishment of short‐term activated T cells as described under in vitro culture of lymphocyte populations. Tumor cells were resuspended in RPMI1640 supplemented with 2 mM L‐glutamine, 25 mM Hepes, 100 U/mL penicillin, 100 µg/mL streptomycin, 10% FCS (complete medium) plus 10% autologous ascites supernatant and cultured in 75 or 175 cm^2^ culture flasks to propagate tumor cells.

PBL were isolated from the leukocyte concentrates or from heparinized blood of patients by Ficoll‐Hypaque density gradient centrifugation, washed, and resuspended in complete medium.

### 2.3 Flow cytometry

A total of 1 × 10^6^ PBL, TAL, and TIL were stained by multicolor flow cytometry approach to distinguish between diverse γδ T cell subsets within different CD45^+^ leukocyte populations. Directly conjugated mAbs included anti‐CD45 clone 2D1, anti‐pan TCRγδ clone 11F2 (both BD Biosciences), anti‐CD56 clone CMSSB (Thermo Fisher Scientific, Langenselbold, Hesse, Germany), anti‐CD3 clone SK7, anti‐CD4 clone OKT4, anti‐CD8 clone SK1 (all three BioLegend, San Diego, CA, USA), anti‐Vδ1 clone REA173 (Miltenyi Biotec), anti‐Vδ2 clone B6 (BD Biosciences), anti‐Vγ9 clone 7A5,^22^ anti‐Vγ2,3,4 clone 23D12,^23^ anti‐Vγ3,5 clone 56.3,^24^ and corresponding isotype controls (BD Biosciences or BioLegend).

To determine cytokine expression in different γδ T cell subsets within PBL, TAL, or TIL, surface stainings of T cells with anti‐CD3 clone SK7, anti‐pan TCRγδ clone 11F2, anti‐CD4 clone OKT4, and anti‐CD8 clone SK1 mAbs were combined with intracellular IFN‐γ, IL‐4, IL‐9, IL‐10, IL‐17, granzyme A/B and TNF‐α mAb stainings in unstimulated cells or after stimulation of the cells with phorbolester 12‐*O*‐tetradecanoylphorbol‐13‐acetate (TPA) and calciumionophore ionomycin for 6 and 20 hr (in the presence of monensin for the last 4 hr). For intracellular staining, 5 × 10^5^ cells were washed with staining buffer, fixed and permeabilized with the Cytofix/Cytoperm kit (BD Biosciences). Thereafter, cells were washed twice with Perm/Wash by centrifugation and stained with fluorochrome‐conjugated anti‐cytokine or anti‐granzyme mAb for 30 min, washed and measured.

To distinguish between leukocytes and tumor cells in 2–5 × 10^5^ TIL and TAL, cells were stained with mAb detecting surface antigens as follows: anti‐CD45 clone 2D1 (BD Biosciences), anti‐HER‐2 clone 24D2, and anti‐epithelial cell adhesion molecule (EpCAM) clone REA‐125 (both from Miltenyi Biotec) followed by intracellular staining with anti‐pan‐cytokeratin clone CK3‐6H5 mAb (Miltenyi Biotec).

All samples were analyzed on a LSR‐Fortessa flow cytometer (BD Biosciences) using CellQuestPro, Diva or FlowJo software.

### 2.4 Characterization of tumor cells by in situ hybridization (FISH technique) and treatment with cisplatin

Tumor‐specific chromosome diversifications of primary tumor cells were analyzed by using the tricolor probe TERC (3q26)/MYC (8q24)/SE7 TC (#KBI‐10704, Kreatech/Leica, Wetzlar, Germany) for fluorescence in situ hybridization (FISH) as described.^25^ Portions of cells deviate from diploid Fish‐signal pattern and overtop the cut‐off are recognized as tumor cells. Briefly, cells were cultured directly on 2‐well Lab‐Tek chamber slide (Thermo Fisher Scientific) overnight to reach a continuous adherence of approximately 70%. Thereafter, the slides were denatured in denaturation solution (2× SSC; 0.5% Igepal) for 15 min at RT and dehydrated in an ascending ethanol series (70%, 90%, 100% ethanol) for each 1 min and air dried. The hybridization process was started without prior fixation of the cells. Therefore, 7 µl FISH probe was added to each sample cover‐slipped (18 × 18 mm) and sealed with rubber cement. Subsequently, the samples were placed at 72°C for denaturation for 10 min and placed in a humidified chamber at 37°C overnight. The slides were placed in SSC‐wash buffer (2× SSC; 0.1% Igepal) for 2 min at RT, followed by 2 min in post‐SSC wash buffer (0.4 × SSC; 0.1% Igepal) at 72°C and 1 min in SSC‐wash buffer. Again, the samples were then dehydrated in an ascending ethanol series for 1 min each and subsequently air dried. Cells were counterstained with DAPI. Signals were visualized by fluorescence in situ microscopy on a Zeiss axioplan microscope using filter combinations for DAPI, FITC, spectrum gold, and 7‐Diethylaminocoumarin‐3‐carboxylic acid. Aneuploidy was detected in 60–100% of cells in each investigated primary ovarian tumor sample, which classified them together with a high expression of HER‐2 and EpCAM as cancer cells.

Depending on the ovarian tumor cell, 7000–15000 cells (KI‐OC‐1, KI‐OC‐11, and KI‐OC‐12) were seeded in 96‐well plates and treated with cisplatin (obtained from the Clinic Pharmacy Services, UKSH, Campus Kiel, Germany) at a concentration of 1 mM (solved in NaCl) at working concentrations of 1–500 µM or NaCl as a control for 48 hr. Cell viability was determined as relative fluorescence units (RFU, 400_Ex_/505_Em_) using CellTiter‐Fluor Cell Viability Assay (#G6080, Promega, Mannheim, Germany). Therefore, a microplate‐reader (Infinite 200, Tecan, Crailsheim, Germany) was used. Dose‐response curves of normalized values were constructed using GraphPad Prism.

### 2.5 In vitro culture of lymphocyte populations

PBL, TAL, or TIL were cultured in complete medium to establish short‐term T cell lines. To expand Vγ9 γδ T cells, cells were stimulated with 2.5 µM of aminosbisphosphonate (n‐BP) zoledronic acid (Novartis, Basel, Switzerland). To expand Vδ1 γδ T cells or αβ T cells, 24‐well plates were coated with 100 µL of 0.5 µg/mL anti‐Vδ1 TCR mAb clone R9.12 (Beckman Coulter, Krefeld, Germany) or 0.5 µg/mL anti‐CD3 mAb clone OKT3 (Orthoclon, Janssen‐Cilag, Neuss, Germany) overnight at 4°C. After washing the wells, 10^6^ PBL, TAL, or TIL/well were cultured with a final concentration of 1 µg/mL anti‐CD28 mAb clone CD28.2 (BioLegend). Because resting γδ T cells produced only low amounts of IL‐2 after initial stimulation, 12.5 U/mL rIL‐2 (Novartis) were added every 2 d over a culture period of 14 d. After 2 wk, most γδ T cell lines had a purity between 60% and 99% γδ T cells. γδ T cells with a purity <98% were labeled with anti‐TCR αβ mAb clone IP26 (BioLegend) and subjected to magnetic separation to deplete remaining αβ T cells. αβ T cells were labeled with either anti‐CD4 mAb clone OKT4 (BioLegend) or anti‐CD8 mAb clone SK1 (BioLegend) and subjected to magnetic separation to deplete CD4 or CD8 αβ T cells.

To analyze the functional capacity of the different Vγ9‐expressing γδ T cell subsets, zoledronic acid expanded Vγ9‐positive γδ TILs were stained with anti‐pan TCRγδ clone 11F2 (BD Biosciences), anti‐Vγ9 clone 7A5,^22^ anti‐Vδ2 clone 123R3 (Miltenyi Biotec), and anti‐Vδ1 clone REA173 (Miltenyi Biotec) and sorted with a FACSAria (BD Biosciences).

### 2.6 Generation of bispecific antibodies

The bsAbs [HER2xCD3] and [(HER2)_2_xVγ9] as well as the control constructs [CD19xCD3] and [(HER2)_2_xCD89] were generated as published previously.^12^, ^13^ Briefly, Lenti‐X™ 293T cells were transfected with corresponding expression vectors coding for the bispecific single chain fragment variables (bsscFv) [HER2xCD3] and [CD19xCD3] or the tribodies [(HER2)_2_xVγ9] and [(HER2)_2_xCD89] using the calcium phosphate technique including 5 mM chloroquine. [HER2xCD3] and [CD19xCD3] molecules were purified by affinity chromatography using nickel‐nitrilotriacetic acid (Ni‐NTA) agarose beads (Qiagen, Hilden, Germany). Heterodimeric molecules [(HER2)_2_xVγ9] and [(HER2)_2_xCD89] composed of a light chain and a heavy chain derivative and tagged with a C‐terminal hexa‐histidine motif were purified from supernatant by two successive steps of affinity chromatography using CaptureSelect Fab kappa affinity matrix (BAC B.V., Naarden, Netherlands) and nickel‐nitrilotriacetic acid (Ni‐NTA) agarose beads (Qiagen) as described earlier.^12^, ^13^, ^14^ Purity and integrity of bsAbs were verified by capillary electrophoresis using an Experion automated electrophoresis system (Bio‐Rad, Kabelsketal, Germany) and size exclusion chromatography.

### 2.7 Real time cell analyzer (RTCA)

Cytotoxicity against adherent autologous cancer cells was analyzed by an RTCA (X‐Celligence, ACEA Biosciences, Inc., San Diego, CA, USA) in triplicates as described elsewhere.^13^, ^14^, ^16^, ^17^ A total of 5000 to 7500 adherent tumor cells/well in complete medium were added to 96‐well micro‐E‐plate to monitor the impedance of the cells via electronic sensors every 5 min for up to ∼24 hr. After ∼24 hr, medium with or without previously titrated saturating concentrations of bsAb (1 µg/mL bsscFv [HER2xCD3] or tribody [(HER2)_2_xVγ9] or control constructs) or autologous PBL, TAL, TIL or short‐term activated T cells together or not with the indicated 12.5 to 50 U/mL IL‐2 were added to the RTCA‐single plate (SP) assay (X‐Celligence, ACEA Biosciences, Inc.). The impedance of the cells is expressed as an arbitrary unit called cell index (CI), which reflects changes in cellular parameters such as morphological changes (e.g., adherence, spreading), cell proliferation and cell death. Because the initial adherence in different wells can differ slightly, the CI was normalized to 1 after having reached the linear growth phase and before the addition of constructs or suspended cells. When effector cells induced lysis of the tumor cells, the loss of impedance of tumor cells is shown as decrease of the normalized CI. As positive control for killing, tumor cells were treated with a final concentration of 1% Triton X‐100. For the precise analysis of cytotoxicity, the cells were monitored every minute for the indicated time points. By using the RTCA software (version 2.0.0.1301 Copyright© 2004–2012 ACEA Biosciences, Inc.) the raw data files were exported to Microsoft Excel (version 14.0.7128.5000 [32 bit]) for further calculation and described as follows. The mean of Triton‐X‐100 samples was calculated and defined as 100% lysis after 4 and 10 hr after addition of T cells. The ratio of each sample to spontaneous lysis of tumor cells alone was calculated and the ratio was normalized to maximal inducible lysis by Triton‐X‐100.

## 3 STATISTICAL ANALYSIS

The statistical analysis was assessed by using Graph Pad Prism (Graph Pad Software, Inc., La Jolla, CA, USA). The normal distribution assumption was analyzed by the Shapiro‐Wilk normality test. For parametric data of nonmatched datasets, 1‐way ANOVA was used followed by Tukey's multiple comparison test or Dunnett's multiple comparison test. Nonparametric data of non‐matched datasets were analyzed by Kruskal‐Wallis multiple comparison test followed by Dunn's multiple comparison test or by Mann‐Whitney test. All statistical tests were 2‐sided and the level of significance was set at 5%. All appropriate tests were indicated in the figure legends.

## 4 RESULTS AND DISCUSSION

### 4.1 Immune profiling of tumor‐derived T cells in comparison to autologous peripheral blood T cells of ovarian cancer patients

Recently published genome‐wide molecular signatures of a broad range of human tumors revealed a prognostic significance of γδ T cells infiltrating in ovarian tumors.^26^ Because selective targeting of γδ T cells to tumor cells by bsAb has an obvious advantage,^13^, ^16^ we were interested to examine the distribution of the different γδ T cell subsets within the tumor. A detailed knowledge of the distribution allows an assessment of the development of further bsAb selectively targeting γδ T cell subsets to the tumor.

In this study, we demonstrated a slight increase of the CD3 γδ T cell percentage within TIL compared to the PBL of the same donors. Interestingly, the γδ T cell percentage within TAL was more drastically increased in comparison to γδ T cells of the blood or tumor‐tissue of these donors (Fig. 1A). Regarding the mainly described γδ T cell subsets, we observed an increase of Vδ1 T cells within the TAL and TIL compared to the PBL, which is comparable to the increase of CD8 αβ T cells of the same donors (Fig. 1A). The distribution of CD8‐ and CD4‐positive αβ T cells were calculated within the CD3 T cells by excluding pan γδ T cells. Similar to CD4 αβ T cells, the percentage of Vδ2 T cells significantly decreased in TAL and TIL compared to PBL. Although the percentage of CD4 αβ T cells and Vδ2 γδ T cells is reduced at the tumor site, the cells are still present in the tumors.

Figure 1
**Analysis of T lymphocyte subsets within blood, ascites, and tumor tissue of ovarian cancer patients**. (**A**, **B**) To determine the relative percentage of the different T cell subsets cells within peripheral blood (PBL), tumor‐ascites lymphocytes (TAL) and tumor‐infiltrating lymphocytes (TIL), ex vivo isolated cells of ovarian cancer patients (*n* = 31) were stained with anti‐CD45, anti‐CD3, anti‐pan γδ TCR, anti‐Vδ1, anti‐Vδ2, anti‐Vγ9, anti‐Vγ2,3,4, anti‐Vγ5, anti‐CD4, and anti‐CD8 mAbs as indicated and analyzed by LSR‐Fortessa. A gate was set on lymphocytes based on the side scatter properties and CD45‐positive leukocytes to analyze γδ T cells within leukocytes. To distinguish between CD4 and CD8 αβ T cells a gate was set on CD3‐positive cells excluding CD3/pan γδ TCR‐positive cells. For discrimination between Vδ1 and Vδ2 and the different Vγ‐expressing subsets within the CD3/pan γδ TCR‐positive cells, a gate was set on CD3/pan γδ TCR‐expressing T cells. The gating strategy is shown with PBL of one patient. (**A**) Each symbol presents the data of one donor, and the lines in the boxes represent the median value of different independent experiments. Normal distribution assumption of all T cell subsets was analyzed by Shapiro‐Wilk normality test. Based on normal distribution assumption of nonmatched Vδ2, CD4, and CD8 T cells, statistical comparison was done parametrically by using 1‐way ANOVA followed by Tukey's multiple comparison test. Nonparametric, nonmatched datasets of Vδ1 and γδ T cells were analyzed by Kruskal‐Wallis test followed by Dunn's multiple comparison test. Significances are shown as *P* value; * = *P* < 0.05, ** = *P* < 0.01, *** = *P* < 0.001, and **** = *P* < 0.0001 (**B**) Pie diagrams present the mean of the relative percentage of Vγ9Vδ2 γδ T cells (black) and Vγ9‐expressing‐ (dark gray), Vγ2, 3, or 4‐expressing‐ (light gray), or Vγ5 or 8‐expressing‐ (white) Vδ1 and Vδ1/Vδ2‐negative γδ T cells (*n* = 31). Based on the failure of an anti‐Vγ8 TCR mAb the percentage of these cells were calculated on the basis of all other known Vγ‐expressing γδ T cell subsets

**Figure:**
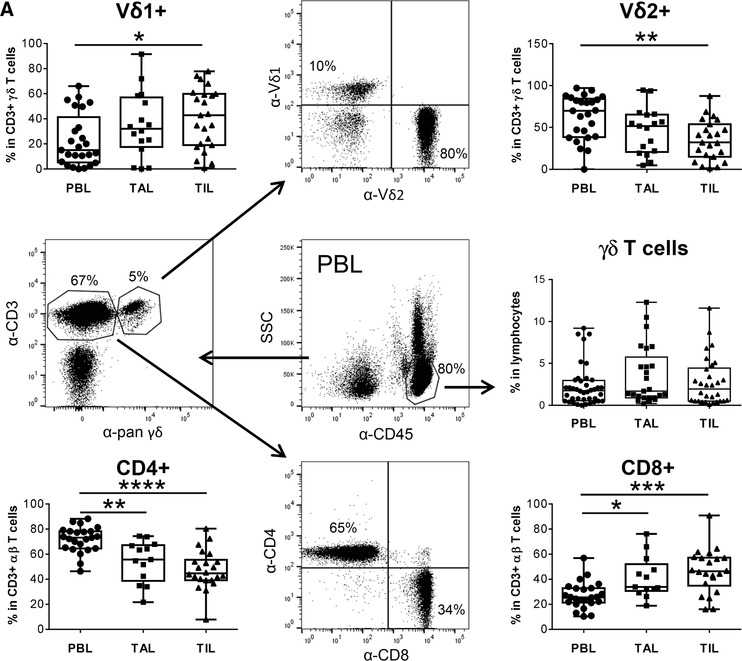

**Figure:**
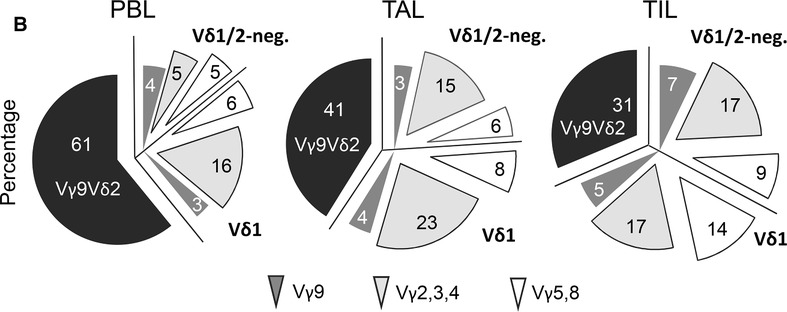

A detailed analysis of the different TCR Vγ chains with our various mAb revealed that the percentage of all Vδ2 T cells that co‐express Vγ9 is reduced at the tumor site (Fig. 1B). The percentage of Vγ9Vδ2 T cells in the PBL of ovarian cancer patients is comparable to the percentage in age‐matched healthy donors (data not shown). Interestingly, we observed an enrichment of Vδ2‐negative T cells (Vδ1 and probably Vδ3) co‐expressing Vγ9 within TAL and TIL compared to PBL of advanced ovarian cancer patients, a subpopulation that is not described within TAL and TIL so far. Beside the Vγ9 chain, the residual Vγ chains were co‐expressed by the Vδ1 as well as with Vδ1/Vδ2‐negative (most likely Vδ3) T cells with a strong enrichment of γδ T cells expressing Vγ2, 3, or 4 within the TAL and TIL (Fig. 1B).

Taken together, our results investigating ovarian cancer patients revealed an inversion of the Vδ1/Vδ2 ratio and an enhancement of Vδ1/Vδ2‐negative γδ T cells co‐expressing mainly the Vγ2, 3, or 4 chain.

### 4.2 Characterization of tumor cells and their cisplatin sensitivity

Immune profiling of tumor‐infiltrating cytotoxic T cells revealed the expression of co‐inhibitory receptors and an exhausted phenotype as described by others.^27^, ^28^

**Figure 2:**
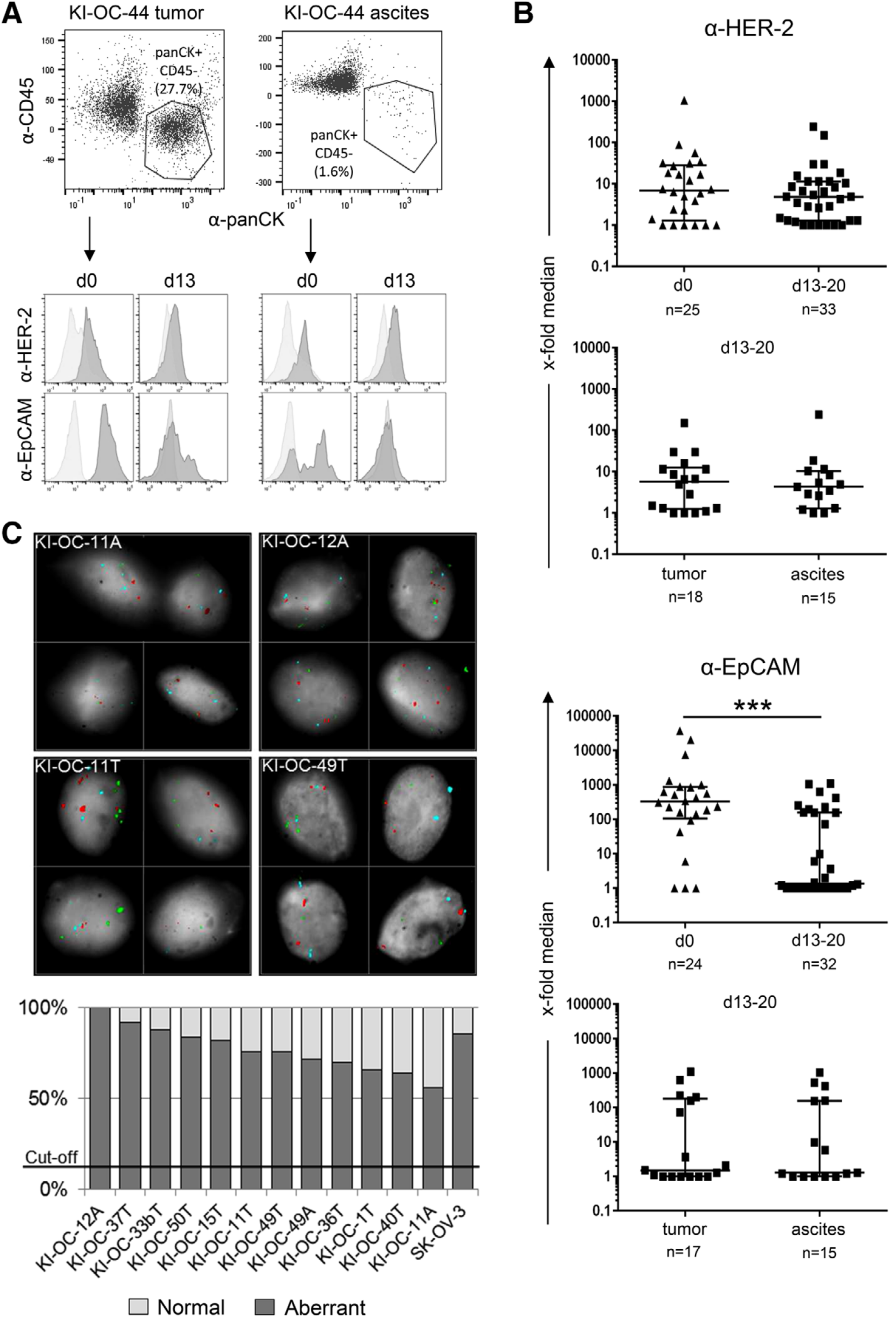
**Characterization of tumor cells from tumor tissue and ascites at d0 and d13‐20 after culture**. (**A**) A representative gating strategy for flow cytometric analysis of pan‐cytokeratin^+^ (α‐pan CK) and CD45‐negative (α‐CD45) tumor cells out of tumor tissue and ascites from ovarian cancer patients (dot plots) is shown. Histograms are showing HER‐2 (α‐HER‐2) or EpCAM (α‐EpCAM) expression (dark gray) compared to isotype‐control (bright gray) at day 0 (d0, immediately after ex vivo isolation) or after 13 d of culture (d13). (**B**) Flow cytometric analysis of HER‐2^+^ (upper two panels) and EpCAM^+^ (lower two panels) tumor cells from ovarian cancer cells directly after ex vivo isolation (d0) and after 13–20 d of culture are presented. In the second panel HER‐2‐ and EpCAM expression are shown on cultured tumor cells derived from tumor tissue (tumor) compared to ascites derived tumor cells (ascites). The median fluorescence intensity was calculated as an *x*‐fold increase in relation to the staining with isotype control. As the majority of the samples did not follow a normal distribution (Shapiro‐Wilk normality test), Mann‐Whitney test was applied for nonparametric, nonmatched datasets. Significances are presented as *P* value *** = *P* < 0.001. Because these values do not follow a standard deviation the quartiles for each dot plot have been added. (C) FISH technique of cultured ovarian cancer tumor cells derived from ascites (**A**) and tumor material (T). Hybridization was performed with the tricolor probe (Kreatech) for 3q26 (hTERC, red), 8q24 (C‐MYC, green), and chromosome 7 centromere (SE 7cen, blue). The pictured cells show increased copy numbers (>2) defined as aberrant (Original magnification ×60). Lower panel shows the results of all tested cultured tumor cells. Samples showing an increased percentage (cut‐off >13%) of nuclei with an aneuploid signal pattern (non‐2‐2‐2, aberrant) were defined as tumor cell line

Here, we were interested in the cytotoxic capacity of ex vivo isolated TIL against autologous ovarian cancer cells and the modulation of cytotoxicity by bsAb. Therefore, we isolated tumor cells and TIL out of tumor tissue and ascites from high‐grade serous ovarian carcinoma patients and characterized them directly after isolation and several days after their propagation. To distinguish between TIL and tumor cells, we stained the cells with a 4‐color panel. As shown in Fig. 2A, CD45 was expressed by leukocytes and pan‐cytokeratin by tumor cells, and this discrimination was used to set a gate on pan‐cytokeratin‐expressing tumor cells and to further characterize the expression of tumor antigens such as HER‐2 and EpCAM (Fig. 2A). HER‐2 and EpCAM are mainly expressed on tumor cells and suggested as suitable tumor associated antigens. Interestingly, 72.4% of the ex vivo isolated ovarian tumors expressed HER‐2 and 88% of them EpCAM, analyzed by flow cytometry (Fig. 2A and B). The propagation of the tumor cells over 13–20 d resulted in a decrease of leukocytes due to an appropriate stimulus (data not shown). HER‐2 expression was slightly decreased, but stably expressed after culturing the tumor cells over 13 to 20 d, whereas EpCAM was significantly decreased after culture. The *x*‐fold of the median fluorescence of HER‐2 and EpCAM was nearly comparable in tumor cells out of the tumor tissue compared to ascites (Fig. 2B lower panels).

To confirm the malignant nature of the propagated tumor cells out of tumor tissue (T) and ascites (A) a fluorescence in situ hybridization was performed in 12 different samples and in the established ovarian cancer cell line SK‐OV‐3 (Fig. 1C, Supplementary Fig. S1). We observed an amplification of the genes human telomerase RNA gene (hTERC gene, 3q26, red) and regulator gene c‐MYC (8q24, green) by FISH technique. The centromere of chromosome 7 (SE7 TC, blue) served as euploidy control. To determine the malignancy of the analyzed samples, the percentage of aneuploid nuclei was determined and compared to a previously calculated cut‐off value. All analyzed tumor samples shared aneuploid nuclei that greatly exceeded the cut‐off value, marking them indeed as tumor cells.

An aneuploidy of 60–100% together with a high expression of pan‐cytokeratin, HER‐2 and EpCAM classified the analyzed cells as cancer cells.

**Figure 3:**
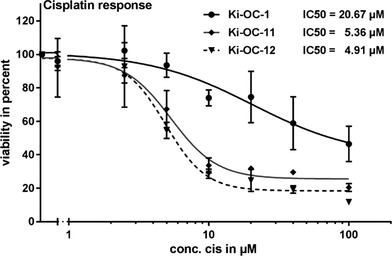
**Impact of cisplatin treatment on cell viability**. Ovarian cancer cell lines (KI‐OC‐1, KI‐OC‐11, and KI‐OC‐12) were treated with the indicated concentrations of cisplatin or the solvent control NaCl. After 48 hr of incubation cell viability was measured using the CellTiter‐Fluor Cell Viability Assay Protocol (Promega). The NaCl‐controls were set to 100%. The mean of three technical replicates ± sd are displayed. IC50 values were determined using GraphPad Prism. KI‐OC‐1 cells (black line, circle) reveal higher resistance to cisplatin compared to KI‐OC‐11 (gray line, diamond) and KI‐OC‐12 cells (dashed line, triangle)

As chemotherapeutic resistance is one of the major clinical challenges in ovarian cancer, we investigated several of the newly established ovarian tumor cells, which were propagated over 20 d and still expressed pan‐cytokeratin, HER‐2, and EpCAM, with respect to their response to chemotherapeutic treatment. Therefore, we incubated the cells with increasing amounts of the chemotherapeutic agent cisplatin for 48 hr and evaluated their IC50 values. Platinum derivates are still the first‐line therapy in ovarian cancer and established ovarian cancer cell lines are usually categorized into a platin‐sensitive and platin‐resistant group. According to the literature, IGROV‐1 and A2780, with IC‐50 values below 5 µM are called platin sensitive whereas the cell line SK‐OV‐3 (IC‐50 > 10 µM) is defined as platin resistant.^29^, ^30^ Based on this categorization the KI‐OC‐1 cells with an IC‐50 of ∼21 µM are rather platin resistant whereas KI‐OC‐11 and ‐12 (IC50: ∼5 µM) belong to the group of sensitive (or intermediate sensitive cell lines; Fig. 3).

### 4.3 Enhancement of cytotoxicity of peripheral blood and tumor‐derived T cells against autologous ovarian tumor cells

A very promising strategy to overcome certain limitations of mAb and occasionally described HER‐2 resistance mechanisms or chemotherapy resistance can be the use of bsAb targeting all CD3 T cells to tumor cells.

To analyze the efficacy of our recently designed bsscFv [HER2xCD3] on the cytotoxicity of different T cell subsets (CD4, CD8, Vδ1, and Vδ2) out of blood (PBL), ascites (TAL), and tumor tissue (TIL) against our newly propagated ovarian tumor cells, we cocultured autologous cell populations and determined cytotoxic activity of T cell subsets by an RTCA (Fig. 4). A limited amount of the different ex vivo isolated T cell subsets out of the material obtained from cancer patients, prompted us to generate short‐term polyclonally expanded T cell subsets to obtain a first impression of their cytotoxic activity against autologous tumor cells and to distinguish between these different T cell subsets. Different effector/target (E/T) ratio of Vδ1 T cells isolated out of PBL of donor OC12 cocultured with autologous KI‐OC‐12 tumor (T) cells revealed an impressive cytotoxic activity (Fig. 4A (a)), which could be further enhanced by the addition of bsscFv [HER2xCD3] (Fig. 4A (b)). Similar results were obtained by using CD8 αβ T cells or Vδ2 T cells of donor OC1 against KI‐OC‐1 tumor cells (Fig. 4A (c)). CD4 αβ T cells isolated out of PBL or TAL did only slightly induce cytotoxic activity against autologous tumor cells (data not shown, Fig. 4A (d)). In contrast to cytotoxic T cells (CD8, Vδ1, and Vδ2) isolated out of PBL of cancer patients OC12 and OC1, T cells isolated out of the ascites (TAL) from the same donors showed an reduced capacity to lyse autologous tumor cells (Fig. 4A (d), (f)), which can be drastically enhanced in the presence of bsscFv [HER2xCD3] (Fig. 4A (e) and (f)). Interestingly, the capacity of cytotoxic T cells isolated from the tumor tissue (TIL) against autologous tumor cells was impressive compared to the TAL subsets (Fig. 4A (g) and (i)); however, TIL cytotoxicity can, similar to TAL cytotoxicity, be further enhanced by bsscFv [HER2xCD3] (Fig. 4A (h) and (i)). BsscFv [HER2xCD3] alone (Fig. 4A (c)) and control constructs such as bsAb [HER2xCD89] did not trigger target cell lysis (Fig. 4A (g)).

Figure 4
**Enhancement of short‐term expanded T cell‐mediated lysis of autologous ovarian tumor cells by bsscFv [HER2xCD3]**. (**A**) A total of 5 to 7.5 × 10^3^ indicated ovarian tumor cells (propagated out of tumor tissue [T] or ascites [A]) per well were cultured in complete medium overnight (light green lines). Impedance of these adherent tumor cells expressed as CI was analyzed in 5 min steps over ∼24 to 25 hr. After reaching the linear growth phase, tumor cells were cocultured with medium (light green line) or different short‐term expanded autologous T cell subsets (Vδ1, Vδ2, CD8, and CD4) isolated out of peripheral blood lymphocyte (PBL, a‐c), tumor‐ascites lymphocytes (TAL, d‐f), and tumor‐infiltrating lymphocytes (TIL, g‐i) at the indicated E/T ratio with 12.5 IU/mL rIL‐2 in the presence of medium (left panel), 1 µg/mL bsscFv [HER2xCD3] (middle panel), or medium or bsAb (right panel). In addition, 1 µg/mL bsscFv [HER2xCD3] or control constructs such as bsscFv [HER2xCD89] were cultured with tumor cells alone (light blue lines). The loss of tumor cell impedance and thus a decrease of CI correlated with lysis of tumor cells. Lysis of tumor cells was measured after normalization to 1 in 1 min steps for additional 20 hr as indicated and compared to maximal lysis by Triton‐X‐100 (TX‐100, black line). The average of three replicates with sd was calculated (**B**) Several replications of the experiments using different T cell subsets cocultured with autologous ovarian tumor cells of different donors (*n* = 8) in independent experiments were performed. The cytotoxicity of the indicated TIL subsets against the autologous tumor cells in the presence of medium (left panel) or 1 µg/mL bsscFv [HER2xCD3] (right panel) was calculated 4 and 10 hr after addition of TIL subsets. The percentage of specific lysis was calculated by comparing measured samples to control sample (green line) and maximal lysis (black line). Based on normal distribution assumption (Shapiro‐Wilk normality test) of nonmatched samples, statistical comparison was done parametrically by using 1‐way ANOVA followed by Dunnett's multiple comparison test. Significances are shown as *P* value; * = *P* < 0.05

**Figure:**
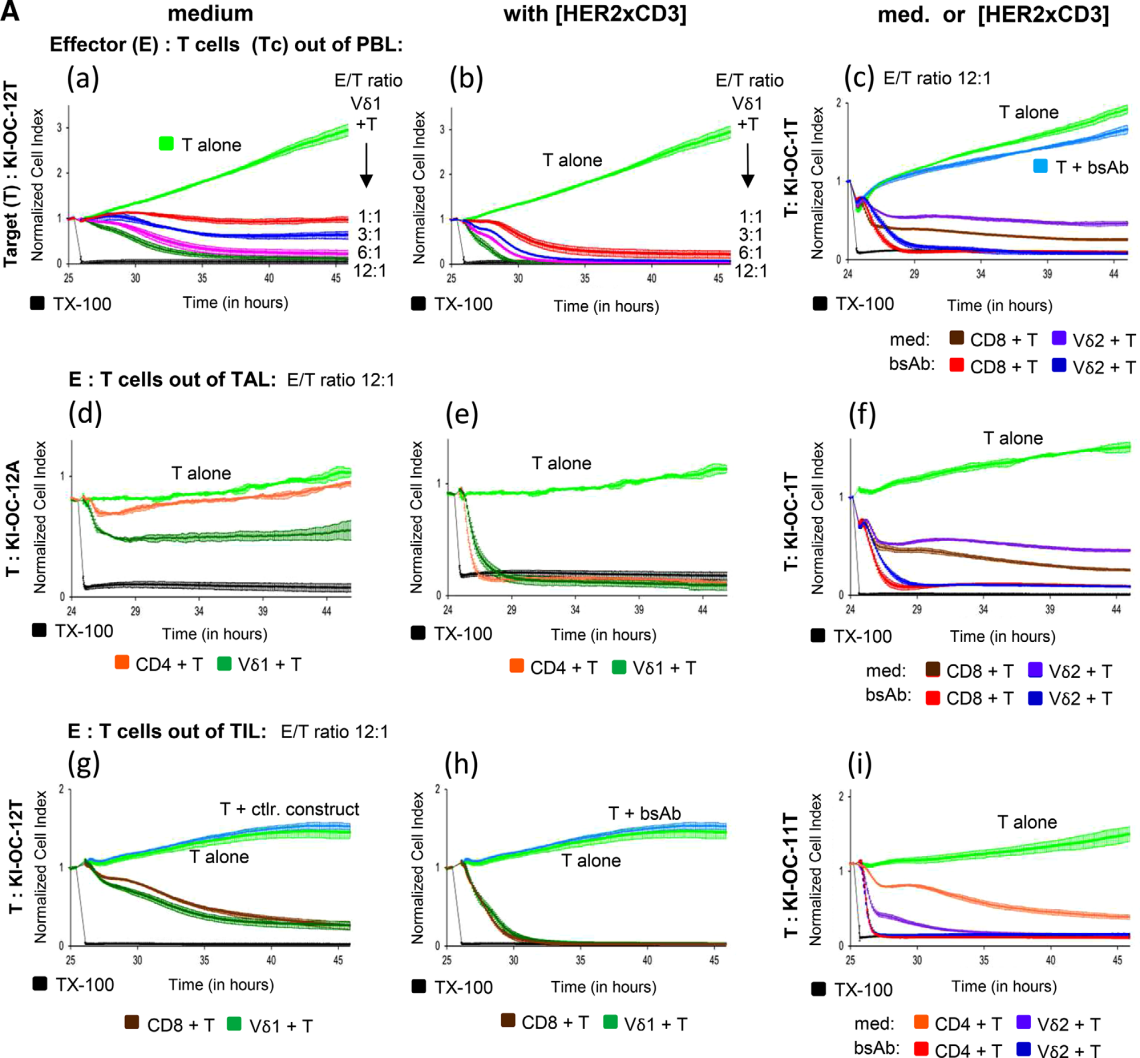

**Figure:**
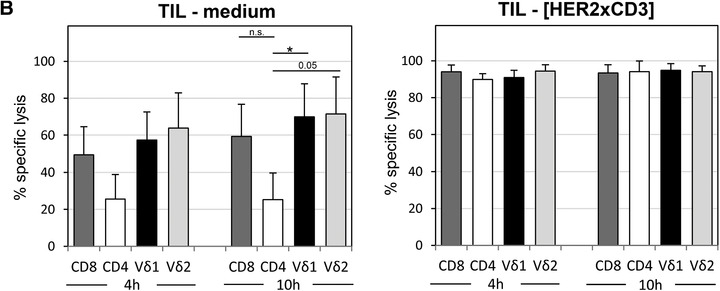

In additional experiments with further pairs of T cell subsets and autologous tumor cells isolated out of tumor tissue (*n* = 8), we confirmed our results (Fig. 4B). We observed a specific lysis of tumor cells by autologous Vδ1 and Vδ2 T cells that is similar or slightly enhanced compared to cytotoxic CD8 αβ T cells after 4 or 10 hr of coculture. As expected, the cytotoxic activity of CD4 αβ T cells was less pronounced. Importantly, bsscFv [HER2xCD3] efficiently enhances cytotoxicity of TIL subsets against HER‐2‐expressing primary ovarian cancer cells (Fig. 4B).

In addition, our results revealed that HER‐2 expressed on high‐grade ovarian tumors can be an efficient tumor antigen for bsAb targeting HER‐2‐expressing ovarian cancer cells to T cells.

Whereas the results with the short‐term expanded T cell subsets are informative when considering an adoptive transfer of T cells in combination with bsAb, they did not reflect possible inhibitory effects by other tumor‐associated immune cells within the TIL due to the selective expansion of the T cell subsets within TIL in vitro. To this end, we considered to apply whole cell population of TIL and TAL in comparison to PBL in further experiments.

### 4.4 Autologous TIL showed a higher cytotoxic activity capacity compared to PBL

**Figure 5:**
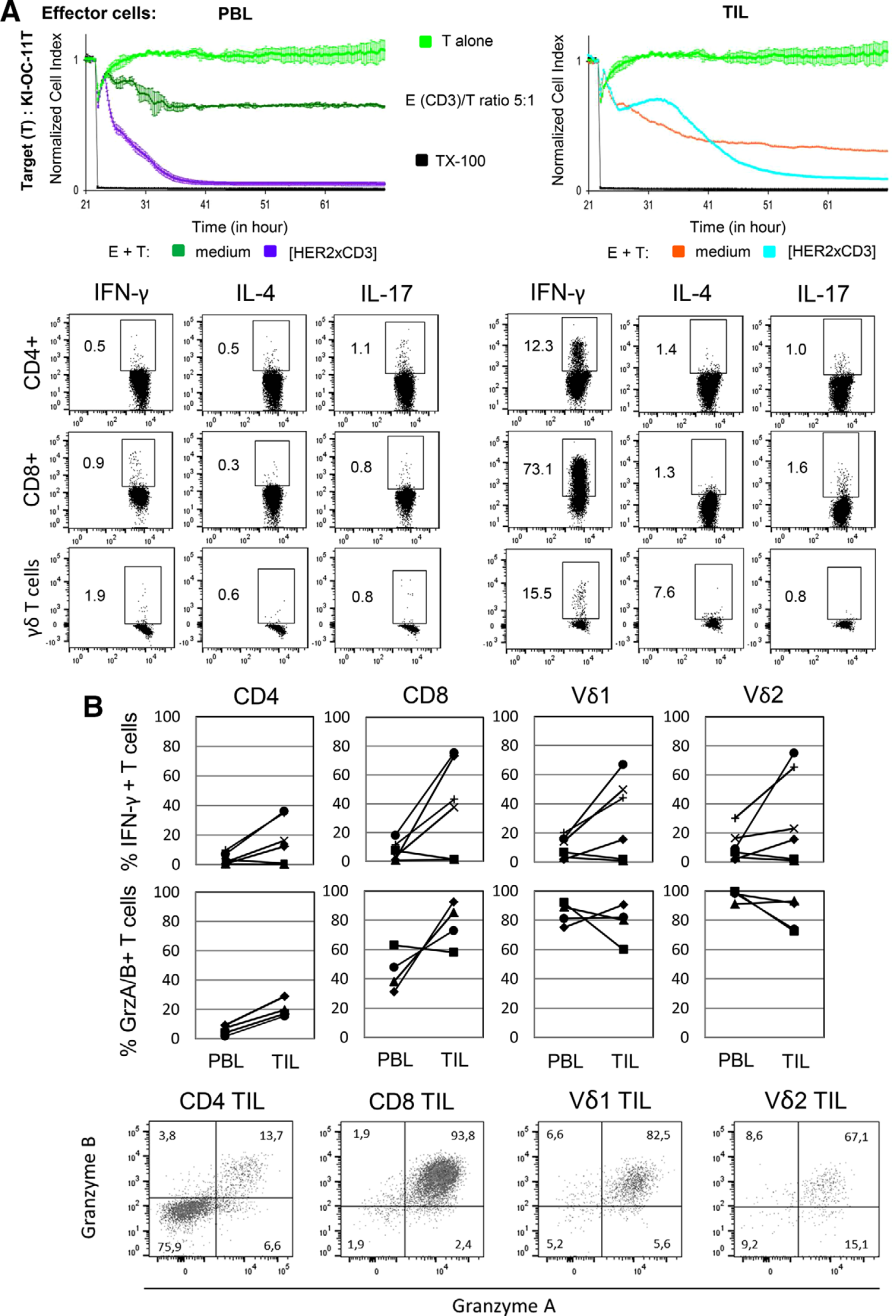
**Effect of bsscFv [HER2xCD3] on PBL‐ or TIL/TAL cytotoxicity against autologous ovarian cancer cells and determination of cytokine production**. (**A**) After culturing 5 × 10^3^ ovarian tumor cells of patient OC11 overnight, cells were cultured without (light green line) or with autologous PBL (dark green line) or TIL (orange line) at an E/T ratio of 5:1 in the presence of 12.5 IU/mL rIL‐2 with medium or with 1 µg/mL bsscFv [HER2xCD3] (violet line for PBL and light blue line for TIL). As controls cells were left untreated (light green line) or treated with Triton‐X‐100 (TX‐100, black line). The cell index (CI) was analyzed in 5 min steps over ∼21 hr and after normalization to 1 (after addition of PBL or TIL ± substances) in 1 min steps for >45 hr as indicated. The average of three replicates with sd is presented for each tumor cell line with or without PBL or TIL. In parallel, relative percentage of intracellular expression of IFN‐γ, IL‐4, and IL‐17 was measured in the indicated T cell subsets of PBL and TIL after surface staining of the indicated epitopes (CD4, CD8, pan γδ TCR) in patient OC11 by flow cytometry after 6 hr of stimulation (**B**) Intracellular expression of IFN‐γ after 6 hr of stimulation (*n* = 6) and granzyme A and B in unstimulated cells (*n* = 4) expressed as relative percentage were analyzed by LSR‐Fortessa. To distinguish between the different T cell subsets, TIL were stained with anti‐CD4, anti‐CD8, anti‐Vδ1, and anti‐Vδ2 mAbs as indicated before intracellular staining. One representative result of four patients is shown with double staining of granzyme A and B as a dot plot graph

Recently, others reported that an inflammatory tumor microenvironment contributes to exhaustion and the expression of co‐inhibitory receptors on cytotoxic T cells.^27^, ^28^ We, therefore, investigated whether the application of bsAb can overcome a suggested exhaustion of cytotoxic T cells within whole TIL population (which can also contain suppressive immune cells). We applied tumor cells initially generated out of the tumor from patient OC11, which revealed an intermediate cisplatin response (Fig. 3) and a high expression of HER‐2.^14^ We cocultured these tumor cells with autologous PBL or TIL ex vivo isolated from patient OC11 after adjuvant therapy followed by three further cycles with carboplatin/paclitaxel and a relapse of the disease. Interestingly, cytotoxic cells within PBL showed a minor capacity to lyse autologous tumor cells than cytotoxic cells within TIL (Fig. 5A, RTCA). A possible explanation of the observed difference might be the activated stage of TIL expressed by an enhanced production of cytotoxic mediators such as granzyme A and B by the T cell subsets within TIL, in contrast to PBL (Fig. 5B, lower panel). Importantly, the application and thus stimulation of the T cell subsets with bsscFv [HER2xCD3] significantly enhances the T cell‐mediated lysis of autologous tumor cells of patient OC11. Interestingly, by comparing the cytokine expression of IFN‐γ, IL‐4, and IL‐17 after stimulation of the T cell subsets, we observed a strong increase of Th1‐cytokine IFN‐γ in the T cell subsets especially by CD8 T cells within the TIL compared to the PBL of the same patient OC11. In contrast, Th2 and Th17 expressions were <5% in the T cell subsets and nearly absent in γδ T cells in patient OC11. Because reports by others demonstrated that the reconstitution of a depressed Th1 response within the tumor is desirable,^31^ we analyzed in further patients whether the stimulation of ex vivo isolated T cell subsets within TIL enhanced their IFN‐γ production. As shown, in Fig. 65B IFN‐γ production was enhanced in T cell subsets within TIL compared to PBL in four further patients out of six analyzed patients. Whereas unstimulated T cell subsets within PBL and TIL of patients did only marginally produce IFN‐γ (data not shown), granzyme A and B are produced without a stimulus in a substantial extent by cytotoxic T cells, but not by CD4 T cells, within TIL (Fig. 5B, lower panel). The abundant granzyme A and B production of Vδ1 and Vδ2 T cells within the PBL, in contrast to CD8 T cells, cannot be further enhanced after stimulation (data not shown) and suggested that γδ T cells are in a preactivated stage already in the blood of the patients.

### 4.5 Impaired cytotoxicity of whole TIL population can be recovered by selective activation of γδ T cells by bsAb

Considering that the polyclonal activation of all T cell subsets by bsscFv [HER2xCD3] could have fatal consequences for instance due to a cytokine storm, we investigated whether it could be beneficial to selectively target γδ T cells by our previously developed tribody [(HER2)_2_xVγ9] instead of all T cells. An enhanced cytotoxicity of Vγ9‐expressing γδ T cells against HER‐2‐expressing tumors grafted into immunocompromised mice was reported by us.^13^ However, our previous studies with adoptive transfer of γδ T cells into human tumor‐bearing SCID‐beige mice could not satisfactorily answer the question whether the tribody [(HER2)_2_xVγ9] can activate γδ T cells within tumor‐infiltrating cells and lyse autologous tumor cells. By coculturing PBL versus TIL or TAL with autologous primary tumor cells, we observed an impaired cytotoxicity that can be drastically enhanced in the presence of tribody [(HER2)_2_xVγ9] at a low E/T ratio (Fig. 6A). For comparable reasons, we additionally activated several of the cultured cells with bsscFv [HER2xCD3], which also drastically enhances T cell cytotoxicity (Fig. 6A). In sum, tribody [(HER2)_2_xVγ9] has a potent capacity to activate γδ T cell cytotoxicity within PBL and TAL or TIL or ovarian cancer patients against HER‐2‐expressing autologous tumor cells.

Figure 6
**BsAb [(HER2)_2_xVγ9] enhances cytotoxic activity of Vγ9 T cells within PBL or TIL/TAL and short‐term expanded Vγ9 T cell subsets against autologous ovarian cancer cells**. (**A**) After culturing 5 × 10^3^ of the indicated ovarian tumor cells propagated out of tumor tissue (T) or ascites (**A**) (light green lines) in complete medium for 23–28 hr, impedance of these adherent tumor cells expressed as cell index (CI) was measured every 5 min. The CI was normalized to 1 shortly before the addition of substances as follows: Triton‐X‐100 to induce maximal lysis (TX‐100, black line), autologous PBL with medium (dark green lines), 1 µg/mL tribody [(HER2)_2_xVγ9] (pink lines) or 1 µg/mL bsscFv [HER2xCD3] (violet lines) or autologous TIL or TAL as indicated with medium (orange line) or with 1 µg/mL tribody [(HER2)_2_xVγ9] (dark blue lines) or 1 µg/mL bsscFv [HER2xCD3] (light blue lines) at the indicated E/T ratio with 12.5 IU/mL rIL‐2. As a control, 1 µg/mL tribody [(HER2)2xV_γ_9] was cultured with tumor cells alone (blue line, upper left panel). CI was then measured every minute for additional 30 hr. The average of triplicates and standard deviation were calculated (**B**) After overnight adherence of 5 × 10^3^ ovarian cancer cells in complete medium, cells were cultured with additional complete medium (light green line), Triton‐X‐100 (TX‐100, black line), short‐term expanded autologous Vγ9Vδ2 or Vγ9Vδ1 γδ TIL at an E/T ratio of 12.5:1 in medium or with the 1 µg/mL tribody [(HER2)_2_xVγ9]. CI was then measured every minute for additional 9 hr. The loss of tumor cell impedance and thus a decrease of CI correlated with lysis of tumor cells. The average of triplicates and standard deviation were calculated

**Figure:**
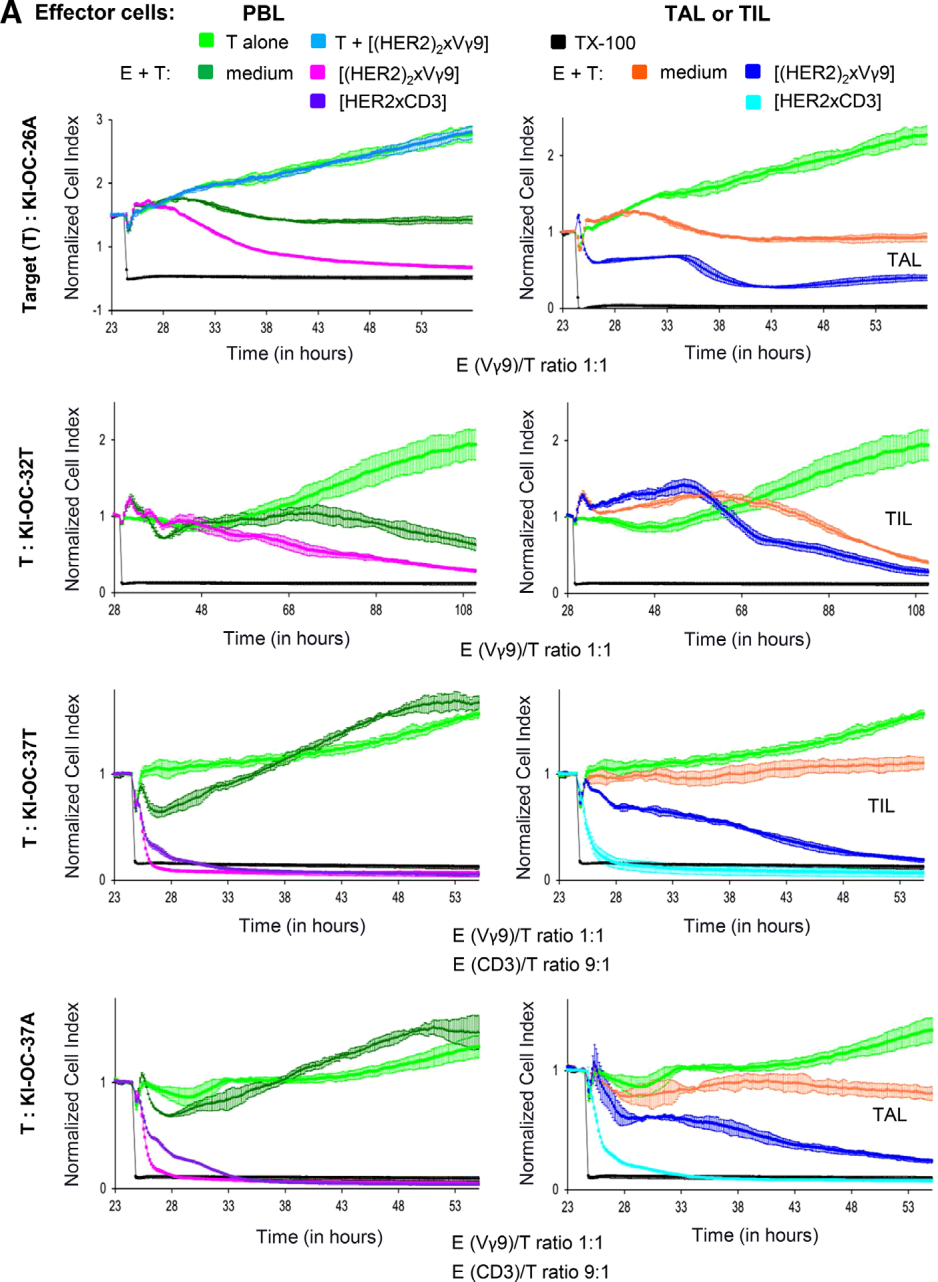

**Figure:**
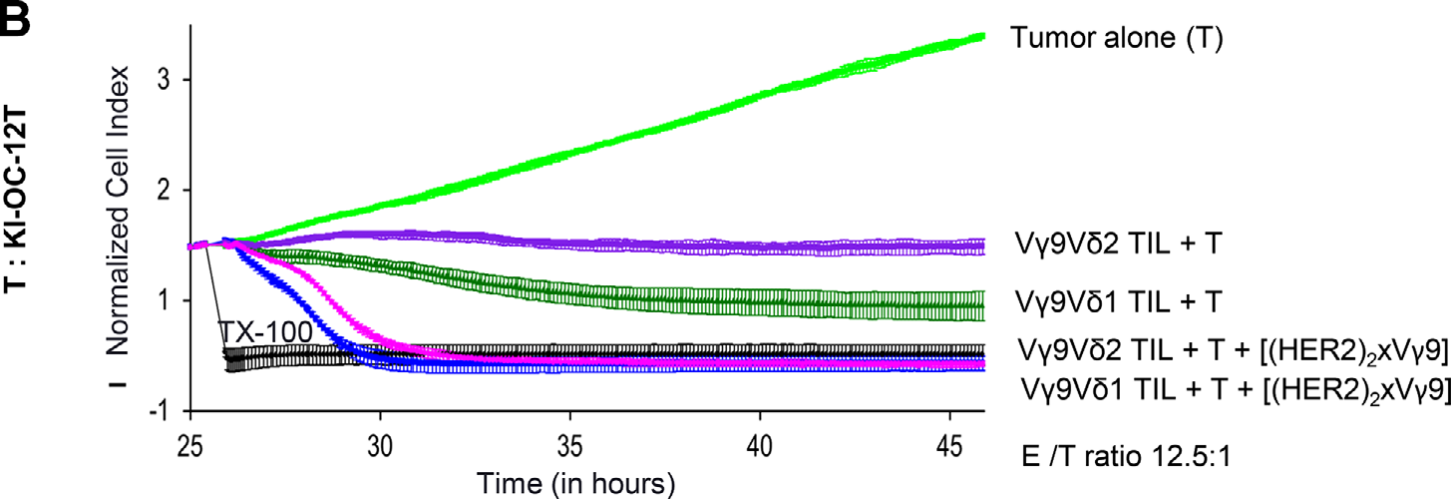

Additionally, we demonstrated that we can target Vγ9‐expressing Vδ2 as well as Vδ1 T cell subsets (sorted out of TIL of the same patient and short‐term expanded due to their limited number) to HER‐2‐ expressing autologous tumor cells. Besides, the cytotoxic capacity of short‐term expanded Vγ9Vδ1‐TIL against autologous tumor cells was superior to Vγ9Vδ2‐TIL in the presence of IL‐2. The addition of tribody [(HER2)_2_xVγ9] induced a complete lysis of tumor cells by both Vγ9 T cell subsets (Fig. 6B).

## 5 DISCUSSION

The high recurrence rate in ovarian cancer disease—even after successful initial surgical and poly‐chemotherapeutic treatment—is a significant problem. Conventional treatment approaches including chemotherapy are often not of long‐lasting effect in the palliative situation.^3^, ^32^ Intratumoral cytotoxic T cells have been correlated with a favorable clinical outcome in ovarian cancer.^33^, ^34^, ^35^ Therefore, monitoring of intratumoral T cell subsets regarding the distribution, phenotype, and their cytotoxic capacity against autologous tumors can give a better assessment of subsequent personalized treatment. While monitoring of phenotypic characterization of human ovarian‐tumor‐infiltrating cells (TIL) as well as prognostic immune‐related risk factors in ovarian cancer has been recently described comprehensively,^28^ results investigating the cytotoxic capacity of the intratumoral T cell subsets against autologous tumor cells are currently missing.

In our study, we expand the phenotypic characterization of the T cell subsets within PBL, TAL, and TIL to the different γδ T cell subsets. We revealed an inversion of the Vδ1/Vδ2 ratio in TAL and TIL compared to PBL of the same patients and an enhancement of Vδ1/Vδ2‐negative T cells within the tumor tissue. We observed Vδ2‐negative γδ T cells expressing Vγ9, which were not described so far. More importantly, besides our phenotypic monitoring, we demonstrated that the impaired cytotoxic capacity of all T cell subsets within TIL and TAL against autologous high‐grade serous ovarian tumor cells expressing HER‐2 can be efficiently enhanced by the application of bsscFv [HER2xCD3] and tribody [(HER2)_2_xVγ9] in vitro.

Interestingly, our data demonstrated a HER‐2 expression in high‐grade serous ovarian tumors analyzed by ex vivo isolated tumor cells. Similar results were obtained by others with different methods including in situ hybridization.^7^ Whereas trastuzumab, a humanized mAb targeting HER‐2, together with taxane‐based chemotherapy is considered as standard of care in (neo‐)adjuvant settings for breast cancer patients,^36^, ^37^, ^38^, ^39^ the efficacy of trastuzumab for ovarian cancer is discussed controversially and needs to be elucidated in more detail.^4^, ^40^, ^41^ Whereas single drug trastuzumab treatment revealed only moderate activity in HER‐2‐expressing ovarian cancer,^4^ the combination therapy of trastuzumab plus chemotherapy are suggested to be more promising as shown for HER‐2‐expressing uterine serous and high‐grade endometrioid tumors.^5^, ^6^, ^42^

Our previously developed bsscFv [HER2xCD3] and tribody [(HER2)_2_xVγ9] 13 efficiently enhance cytotoxicity of PBL, TIL, and TAL against autologous HER‐2‐expressing ovarian cells. One strategy to overcome described trastuzumab resistance can be the commitment of bsAb targeting T cell subsets to HER‐2‐expressing tumor cells.^11^ Regarding bsAb, we made a very important observation during our investigations. T cell subsets within TAL revealed an impaired cytotoxic activity against autologous tumor cells isolated out of ascites compared to TIL against tumor cells isolated out of tumor tissue, which both can be overcome after application of bsAb in vitro. An impaired cytotoxic activity of TAL is suggested to be due to an abundant expression of co‐inhibitory receptors such as programmed cell death‐1 (PD‐1), lymphocyte‐activation gene‐3 (LAG‐3), and cytotoxic T lymphocyte antigen‐4 (CTLA‐4) in comparison to peripheral blood lymphoctes (PBL)^27^ or to an exhaustion of TAL.^28^, ^43^ In contrast, Radestad and colleagues reported that the expression of co‐inhibitory receptors and a downregulation of CD127, which occurs after persistent antigen stimulation in exhausted T cells, was more pronounced in TIL than in TAL, and correlated with a reduced survival.^28^, ^44^ Because our patients analyzed in the functional assay are still alive after a median follow‐up of 11.5 to 44 mo, we cannot draw conclusions to their outcome. Of course, we observed an enhanced expression of PD‐1 on αβ‐ and γδ TIL and TAL; however, PD‐L1 was only expressed on KI‐OC‐1 tumor cells but not on KI‐OC‐11, KI‐OC‐12, and KI‐OC‐15 tumor cells (data not shown). Importantly, the treatment with bsAb can overcome this inhibitory interaction. Another crucial factor is the enrichment of ascites with tumor‐promoting soluble factors, extracellular vesicles and suppressive tumor‐derived immune cells.^43^ We cultured our propagated tumor cells out of tumor tissue and ascites with 10% ascites supernatant, and in several experiments we added 10 to 20% of ascites supernatant, which did not negatively influence the efficacy of the bsscFv [HER2xCD3] or tribody [(HER2)_2_xVγ9].

Recently, we reported that the application of bsscFv [HER2xCD3] and tribody [(HER2)_2_xVγ9] enhanced the granzyme B release of PBL‐derived T cells of healthy donors and pancreatic cancer patients, whereas the intracellular expression of granzyme B was nearly comparable in unstimulated versus bsAb‐activated cells.^13^, ^14^ By comparing the intracellular granzyme A and B expression of ex vivo isolated PBL versus TIL of ovarian cancer patients without stimulation, we observed an enhanced production of both cytotoxic mediators in CD8‐TIL compared to CD8‐PBL, whereas the production in Vδ1 and Vδ2 T cells was similar in PBL and TIL suggesting that γδ T cells in the analyzed ovarian cancer patients are in an pre‐activated stage. Because granzyme A and B production of TIL is already at the highest achievable level, stimulation of these cells with a polyclonal stimulus enhanced slightly further the production of these cytotoxic mediators (data not shown). In contrast to granzyme A and B production, cytokine expression of IFN‐γ, TNF‐α, IL‐4, IL‐9, IL‐10, and IL‐17 was not observed in unstimulated ex vivo isolated T cell subsets of PBL, TIL, and TAL. Interestingly, after polyclonal stimulation, we observed a drastic increase in IFN‐γ and TNF‐α production in four out of six analyzed TIL compared to PBL of the same donors. In our ongoing experiments, we revealed an enhanced IFN‐γ production after stimulation of TIL with bsscFv [HER2xCD3] or tribody [(HER2)_2_xVγ9]. This is in line with the data reported by Datta et al., which demonstrated that the treatment of breast cancer patients with HER‐2‐pulsed dendritic cells restore a depressed Th1 response mediated, for example, by IFN‐γ release.^31^, ^45^

Considering that the polyclonal activation of all T cells by bsscFv [HER2xCD3] can induce a cytokine storm as an undesirable side effect, it would be desirable to activate only γδ T cells. Our results revealed that the cytotoxic activity of all Vγ9‐expressing γδ T cells within PBL and TIL can be enhanced against autologous tumor cells in the presence of tribody [(HER2)_2_xVγ9]. In our further investigations, we will clarify the important question whether the application of bsAb targeting γδ T cells to ovarian tumor cells can overcome the immunosuppressive tumor microenvironment by an in vivo model. Of course, the first‐line therapy of ovarian cancer would be a surgical resection. However, even after a clinical remission, several patients suffer from a relapse of the disease and become refractory to chemotherapy during the course of therapy.^46^ An alternative adjuvant therapy of chemotherapy resistant patients could be the treatment with bsAb. In this context, the report of Lai et al. is very interesting, which demonstrated that small population of cancer stem cells that are responsible for resistance to cancer therapies and tumor maintenance can be efficiently killed by γδ T lymphocytes.^47^

In conclusion, a personalized assessment of phenotypic and functional characterization of the T cell subsets of tumor patients is important to get insights in the anti‐tumor response of tumor‐infiltrating and circulating T cells and to develop new strategies to enhance their cytotoxic activity. BsAb, which selectively targets immune cells to solid tumor cells and enhances their cytotoxicity, provides a valuable tool to analyze the functional capacity of T cell subsets within PBL, TAL, and TIL. Whether bsAb targeting αβ or selectively γδ T cells have the capacity to overcome an immunosuppressive microenvironment, has yet to be investigated in further in vivo studies.

## DISCLOSURES

The authors declare no conflicts of interest.

## Supporting information

Supplemental Information
