# Supplementary material for: Bispecific antibodies enhance tumor‐infiltrating T cell cytotoxicity against autologous HER‐2‐expressing high‐grade ovarian tumors
Source: J Leukoc Biol. 2019 Dec 13;107(6):1081–95. doi: 10.1002/JLB.5MA1119-265R (PMC7318294; doi:10.1002/JLB.5MA1119-265R)
Supplement: Supplementary file 1 — Supplemental Information [file JLB-107-1081-s001.pdf]

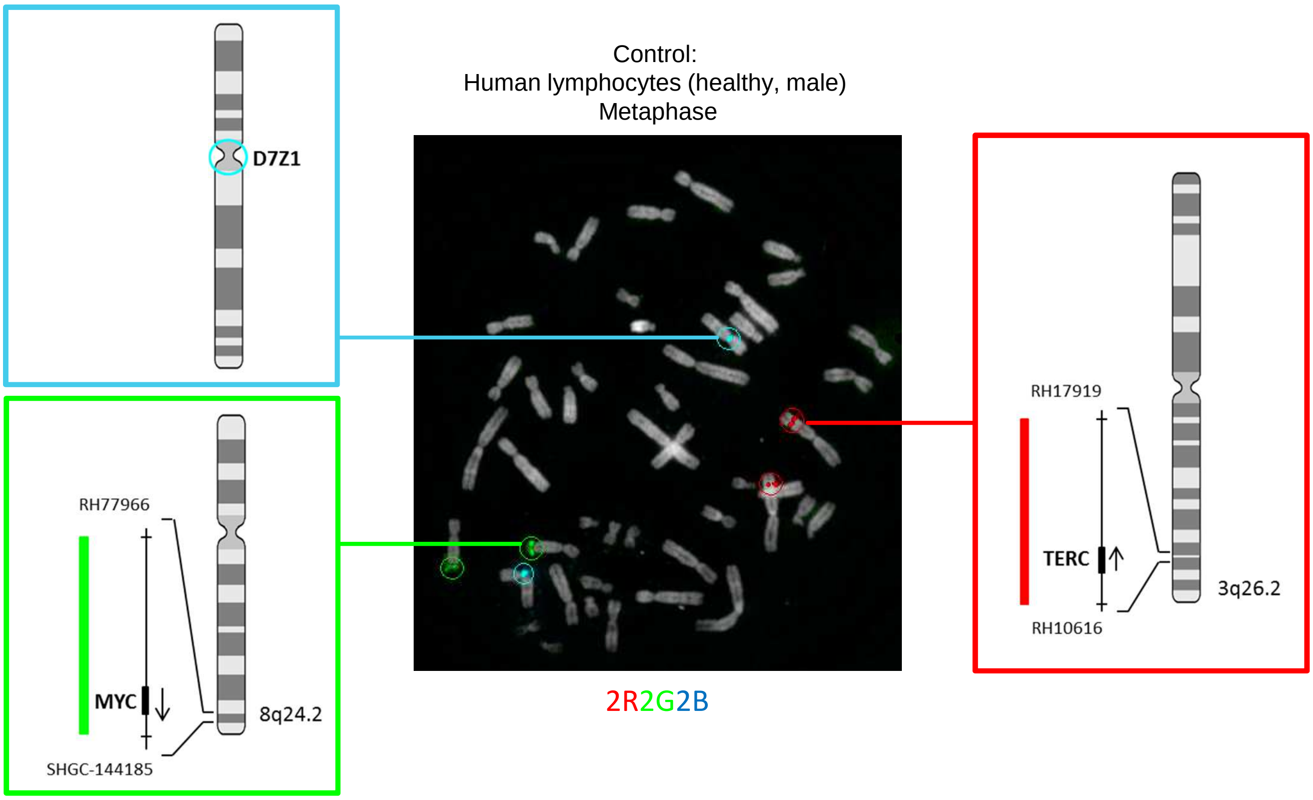

Supplemental Figure 1: Scheme of Kreatech/Leica tricolor probe KBI-10704. Diagrams of normal chromosomes three, eight and seven along with the fluorescence labeled region in our utilized FISH probe. Middle: Exemplary metaphase nucleus of a healthy lymphocyte. There are two distinct signals for each color discernible. Adapted from Leica Biosystems product catalog. (https://www.leicabiosystems.com/fileadmin/biosystems/PDF/95.14492_LBS_AS_Product_Catalog_2017_LR.pdf)
